# Respiratory-swallow assessment protocol for adult dysphagia management

**DOI:** 10.1186/s13104-025-07509-4

**Published:** 2025-11-27

**Authors:** Liza Bergström, Julie Cichero, Mariam Jaghbeer, Anna-Liisa Sutt

**Affiliations:** 1 Division of Neurology, Department of Clinical Sciences, Karolinska Institutet, Danderyd University Hospital, Stockholm, Sweden; 2 Remeo Intensive Care Rehabilitation Center, Stockholm, Sweden; 3 School of Pharmacy, The University of Queensland, Brisbane, Australia; 4 Eat Drink Swallow Safe, Brisbane, Australia; 5 Independent Speech and Language Therapist and Researcher, Amman, Jordan; 6 Speech and Language Therapy, The Royal London Hospital, Barts Health NHS Trust, London, UK; 7 Critical Care Research Group, Institute of Molecular Bioscience, University of Queensland, Brisbane, Australia

**Keywords:** Cervical auscultation, Oropharyngeal dysphagia, Deglutition, Evaluation

## Abstract

**Introduction:**

Cervical auscultation (CA) analyzes respiratory-swallow patterns to enhance the clinical swallow examination. A growing body of research demonstrates CA’s strong psychometric properties for detecting unsafe swallows. However, to date, no standard CA assessment protocol or training resources have been published.

**Objective:**

Extending previous work, the objective is to publish the evidence-based assessment guide used in our earlier research.

**Method:**

Additional qualitative data and recent literature was integrated to update the previously unpublished CA guide. Qualitative content analysis was performed on 71 data texts from 12 CA-trained Speech-Language Pathologists describing CA respiratory-swallow signs that influenced their ratings of six swallow sound recordings. The CA ratings were compared against Flexible Endoscopic Evaluation of Swallowing (FEES).

**Results:**

Qualitative results, describing the swallow and respiratory characteristics of dysphagic swallows, were integrated into the CA assessment guide (Supplementary Material 9). Five critical aspects to CA assessment include: (1) pre-swallow respiratory sounds, (2) swallow sounds, (3) number of swallows, (4) post-swallow exhalation, and (5) post-swallow respiratory sounds. Additional audio sounds (Supplementary Material 1–8) and reference samples are provided (Supplementary Material 9) to facilitate an introductory training resource.

**Conclusion:**

The evidence-based CA guide and training resources are expected to establish clinically important resources for future clinical use and research evaluation.

**Supplementary Information:**

The online version contains supplementary material available at 10.1186/s13104-025-07509-4.

## Introduction

Cervical auscultation involves using a stethoscope or a small microphone attached to the throat to listen to and interpret the sounds of swallowing and associated respiratory patterns at the cervical (neck) level, with the recommended site being immediately inferior to the larynx, laterally [1]. Since the publication of our previous research [2, 3], there has been increased interest in the use of cervical auscultation (CA) for assessing swallowing function [4, 5, 6, 7 and personal communication]. Interest spans the technique itself, the protocols for its application, and the training required to utilise it effectively.

Our most recent study [2] demonstrated strong CA psychometric properties with high to very high sensitivities (>85%) and >90% predictive values to accurately detect unsafe swallows with moderate-to-good reliability of CA, when performed by *trained* Speech-Language Pathologists (SLPs). It is the respiratory-swallow assessment protocol from this study that has been sought after for publication to standardise and improve both clinician training and application of CA in everyday dysphagia management. In alignment with this journal, *BMC Research Notes*, this paper aims to provide extensions to our previously published research [2, 3, 18] by now making this protocol and training resources publicly available. This CA assessment guide is specific to CA clinical assessment using a stethoscope or microphone (not acoustic analysis using AI nor machine learning), which is another rapidly expanding area of CA [7–9].

With increasing clinical interest and a growing body of literature on cervical auscultation [4–11], there is a clear need to standardize protocols for clinical CA assessment. Such standardisation is essential to advance clinical research in a consistent, reproducible manner. This need parallels developments in other areas of dysphagia assessment [12–14] and aligns with professional guidelines from organizations such as Speech Pathology Australia [15] and the American Speech-Language-Hearing Association [16].

By publishing this CA protocol alongside training resources that include audio files and reference samples of both normal and dysphagic swallows, we aim to support the informed and consistent application of CA in clinical practice. This resource is intended to enhance the reliability and clinical utility of CA as a complementary tool to the Clinical Swallow Examination (CSE). Furthermore, it offers a standardised foundation for future validation and research, enabling practitioners and investigators to build upon a shared framework.

## Method

### CA assessment guide

The CA assessment protocol from our 2023 research [2], though not yet published, was reviewed and updated with qualitative analyses and integration of recent literature (as per methodology below). The original protocol demonstrated strong CA psychometric properties with high to very high sensitivities (>85%), and predictive values >90% to accurately detect unsafe swallows. Of note is that the respiratory-swallowing characteristics of *normal* swallows are well documented in the literature [17] and within this protocol. Methods to therefore enhance the protocol included analyzing new CA data from *dysphagic* respiratory-swallowing sequences [18], as described below.

### Qualitative analyses of previous unpublished CA dysphagic respiratory-swallow sequences

Data from the 2014 Bergström et al. study [18], investigated the accuracy of CA to detect if swallow sounds (*n* = 15) were assessed as (a) safe/unsafe and (b) dysphagic (i.e. abnormal/normal) - binary ratings as rated by 13 CA-trained Speech-Language Pathologists (SLPs). Although greater study methodology details can be found within the original article, important details are summarized hereafter. The 15 swallow samples (rated by the 13 CA raters) were recorded simultaneously during a gold standard, Flexible Endoscopic Evaluation of Swallowing (FEES), enabling direct comparison (validity) between CA ratings and the FEES reference test. The FEES assessment included standard penetration-aspiration ratings [19] however not standard residue rating such as the Yale Pharyngeal Residue Severity Rating Scale [20], since this rating scale was not published at the time.

Inclusion criteria for the 13 raters were (i) attendance at a minimum 1-day CA training workshop and (ii) regular use of CA (several times weekly) within their CSE during dysphagia assessments and reviews. This study demonstrated high CA accuracy (sensitivity = 83–95%) and reliability (*r*_s_ = 0.64–0.75). Additional data from this study, not analyzed at the time, included the clinical question: *What were the clinical signs which influenced your CA assessment decision?* It is this previously unpublished qualitative data which is now included in the current paper.

As per FEES assessment, six of the 15 swallows were dysphagic and therefore analyzed using qualitative content analysis. The six swallows were from five heterogenic dysphagic patients – see Table 1 for patient demographics and assessment results, as per original study [18]. The six respiratory-swallow samples were of different consistencies as per the International Dysphagia Diet Standardization Initiative (IDDSI) [21]: two thin fluid (IDDSI-0) swallow recordings, one recording of IDDSI-2 mildly thick fluids, and three samples of IDDSI-4 extremely thick fluids.

**Table 1:** Patient and swallow characteristics as per flexible endoscopic evaluation of swallowing (FEES)

Patient background / diagnosis		FEES assessment (bolus consistency + size) Dysphagia severity ^a^	FEES description (CA swallow clip length)	
1	73 years. Neurosurgery, coiling of peri-collosum aneurysm, subarachnoid haemorrhage. Previous right CVA, residual weakness and spasticity. Depression.	(Thin fluids, 10 ml) *Severe dysphagia*	*Safety*: PAS^b^ 8. *Efficiency*: Severely delayed swallow. 3 swallows, ineffective. Pharyngeal residue post swallows. (CA swallow clip = 20 s)	
2	86 years. Admitted with respiratory distress requiring intermittent CPAP (continuous positive airway pressure), pneumonia (? aspiration pnuemonia). Previous left CVA 3 years earlier, residual right side paresis, aphasia. Dementia.	(Thin fluids, 10 ml) *Severe dysphagia*	*Safety*: PAS 7 *Efficiency*: Severely delayed swallow. 3 swallows, ineffective. Residue post swallows. (CA swallow clip = 22 s)	
3	81 years. Right CVA. Left paresis. Previous history = acromegaly, hypothyroidism, diabetes.	(IDDSI-2 fluids, 10 ml) *Mild dysphagia*	*Safety*: PAS 2. *Efficiency*: Mildly reduced, poor control, coordination. 1 swallow, penetration. Residue post swallow. (CA swallow clip = 6 s)	
4	Swallow 4 = another recording from patient 1. (Same patient, different consistencies.)	(IDDSI-4 fluids, 10 ml) *Severe dysphagia*	*Safety*: PAS 2 *Efficiency*: Poor oral control. Multiple swallows. Residue + + post swallows. (CA swallow clip = 19 s)	
5	76 years. Left CVA, aphasia. Previous TIA.	(IDDSI-4 fluids, 10 ml) *Moderate/severe dysphagia*	*Safety*: PAS 1 (risk for delayed penetration/aspiration secondary to residue) *Efficiency*: Weak pharyngeal phase with multiple swallows required to clear bolus. Residue post swallows. (CA swallow clip = 15 s)	
6	71 years. Parkinsonism. Admitted with respiratory and swallowing difficulties. Past 4–5 months = increased swallowing difficulties, choking, 1 episode of pnuemonia	(IDDSI-4 fluids, 10 ml) *Moderate/severe dysphagia*	*Safety*: PAS 3. Penetration which is not ejected. *Efficiency*: Delayed swallow initiation. 2 swallows, pooling + + between swallows. Residue in larynx and pharynx. (CA swallow clip = 7 s)	

^a^Dysphagia severity rating (a study-specific scale adapted from Aus-TOMS [22]) whereby

Severe dysphagia=Difficulty initiating the swallow consistently and is severely delayed or disordered. Patient will likely require alternative / supplementary nutrition. May be able to safely practice swallowing with small amounts of modified consistencies under supervision

Moderate/severe dysphagia=Moderate oral / pharyngeal impairment with poor bolus control. Swallow initiated but may be inconsistent, very delayed or disordered. Patient may be safe on a limited range of consistencies. Some alternative/supplementary feeding may be required

Mild dysphagia=Mild oral and /or pharyngeal impairment with mild difficulties in bolus control. May demonstrate mild delay or mild pooling. Patient may require some restrictions in range of consistencies however would likely manage most consistencies

^b^Penetration-Aspiration Scale, as per [19]

#### Qualitative analysis methodology

Qualitative data from the CA clinical question (*What were the clinical signs which influenced your CA assessment decision?*) were analyzed using deductive content analysis [23] by authors LB and MJ, initially independently and then together via consensus. Specifically, deductive content analysis was employed since this method aims to test established concepts and pre-defined categories with new data [23]. This methodology also captures nuanced clinical reasoning behind clinical judgements and facilitates the understanding of dysphagia features as per CA assessment.

Meaning units were first identified and coded from each rater’s text. Codes were reviewed and grouped as per categories/concepts – see summary, Table 2. This table also identifies CA rating descriptors as compared to the FEES assessment characteristics, specifically across the Oral Phase and Pharyngeal Phase, and in terms of (i) safety and (ii) efficiency. Insights from this qualitative data were considered for integration into the CA assessment guide to enhance its clinical utility.

### Finalisation of the CA assessment guide

A second review of the initial protocol [2] was conducted by three international CA dysphagia research experts, LB, JC and A-LS (18–29 years of experience). The updated CA assessment guide (Supplementary Material 9, Appendix A) includes new data from the qualitative analyses, as per above methodology (denoted by §), and new CA research published until May 2024. The CA assessment framework reflects contemporary dysphagia assessment concepts including (1) swallow *safety* (penetration and/or aspiration) and (2) swallow *efficiency* (bolus transport through the pharynx).

## Results

### CA assessment data – qualitative analyses

Of the 13 raters, 12 answered the clinical assessment question (*What were the clinical signs which influenced your CA assessment decision?*) for the 6 unsafe/dysphagic swallows (12 × 6 = 72 data texts). One of the raters incorrectly rated one of the swallows as safe, resulting in exclusion of that data point (*n* = 71 valid data texts). A summary of salient codes (dysphagic characteristics) arising from the textual analyses, as per dysphagia assessment categories, are presented in Table 2.

**Table 2:** Flexible endoscopic evaluation of swallowing (FEES) and equivalent cervical auscultation (CA) descriptors (*n* = 71 data texts)

FEES results / dysphagic descriptors	Equivalent CA descriptors
(1) Oral phase Described as: Poor oral control or delayed swallow initiation.	Described by CA raters as: Delayed swallow initiation, pre-swallow spill. Long/difficulty with breath-hold prior to swallow initiation. Poor oral control, tongue pumping / extra oral phase sounds prior to swallow initiation. (Described in 54% of CA ratings, 100% correctly. Note: not all CA raters commented specifically on oral phase features).
(2) Pharyngeal phase	
Safe swallow Safety rating as per Penetration-Aspiration scale [19]	Safety: CA rated as a binary yes/no question. (All raters were required to rate this feature. Correct ratings = 71/72 occasions, 99%)
Efficient swallow Reduced efficiency described as: (i) Multiple swallows with residue post swallow (ii)Weak pharyngeal phase with residue post swallow (residue identified within larynx and/or pharynx)	Reduced Efficiency Swallow and respiratory sound data* described as (with descriptors expanded further upon below): (i) Multiple swallows with residue (characterised by wet respirations during and/or after swallows) (ii) Poor bolus transit through pharynx/prolonged pharyngeal phase (characterised by extra swallows, and/or residue as identified by wet respirations/increased noisy breathing and/or extraneous sounds) (Reduced efficiency described in 70% of ratings, 100% correctly).

*Other CA descriptors (respiratory-swallow sound data) indicating dysphagia:

1. Change in respiratory sounds

Quality of breath sounds: Wet/gurgly respirations (indicative of residue/pooling in larynx/pharynx)

Rate: often increased respiratory rate or change in respiratory rate or respiratory-swallow coordination

2. Respiratory-swallow coordination: indicated by inspiration post swallow and/or long apnoeic period/breath hold with inspiration post swallow.

3. Apnoeic period: long breath-hold or unable to sustain breath holding during swallow sequence (often coupled with inspiration and wet/gurgly sounds post swallow, indicative of residue post swallow).

4. Quality of swallow sounds: Long, dull (or very dull) swallow sounds. Not distinct (discoordinated) swallow sounds. Extraneous swallow sounds.

5. Laryngeal/pharyngeal residue: indicated by change in respiration (often with increased wet respirations), coupled with multiple swallows.

6. Throat-clearing in combination with residue / change in respiratory sounds.

(Examples also provided in supplementary files) NB, Supplementary Material 9 is to be used with the eight additional audio files, with examples of four normal 4 respiratory-swallow sound samples and 4 dysphagic swallow samples.

### CA assessment protocol and reference samples

The CA assessment guide can be found in Supplemantary Material 9, Appendix A. The assessment guide identifies five critical aspects to respiratory-swallow analysis: (1) pre-swallow respiratory sounds, (2) swallow sounds, (3) number of swallows, (4) exhalation post-swallow, and (5) post-swallow respiratory sounds, with specific dysphagic characteristics described. Supplementary Material 9, Appendix B reports on respiratory-swallow descriptions for eight audio reference samples [see Additional Files 1–8]. These reference samples complement the CA assessment guide and are compiled from four normal respiratory-swallow recordings (from author LB, FEES confirmed) and four abnormal swallows collected from previous CA research demonstrating high validity [2]. Supplementary Material 9, Appendix B partners and reflects the eight audio samples [Additional Files 1–8] which include recordings of varying bolus size and IDDSI consistencies [21]. These resources provided in Supplementary Material 9, together with the eight audio samples, provide a small CA training sample and are expected to facilitate correct use of CA to improve clinical implementation. These few training resources, however, do not replace a full training program/package required for CA competency.

## Discussion

This study provides new data, including publication of the CA assessment guide, and CA audio files and reference samples (see Supplementary Material 9, plus additional audio files 1–8). Furthermore, the study facilitates clinical applicability of CA by providing an assessment framework that includes detailed descriptors of normal/abnormal *respiratory*-swallow components, across varying bolus sizes and consistencies. This information, missing from current literature, is ideal for learning the theory of CA and, additionally, provides a sample of training resources as an introduction to skills required for its clinical use.

Cervical Auscultation and dysphagia literature has highlighted the importance of assessing respiratory-swallow coordination; in particular, pre and post swallow respiration [2, 17, 24, 25, 28, 33–35] rather than rating stand-alone swallow sounds. Although the validity of CA (sensitivity and specificity for detecting aspiration) has demonstrated high values [2, 7, 24], the need for published, standard CA criteria and descriptors is warranted [3]. Data from the current study along with the CA assessment guide (Supplementary Material 9) comprehensively describes the respiratory-swallow characteristics as (1) pre swallow respiratory sounds, (2) swallow sounds, (3) number of swallows, (4) post swallow exhalation, and (5) post swallow respiration, which is congruent with previously published literature [17, 24–32].

The current study analyzed qualitative data and identified descriptors indicating difficulties in the oral and pharyngeal phases, including reduced swallow *safety* and *efficiency*. CA raters described changes to respiratory-swallow patterns that were congruent with instrumental findings via FEES. 

For reduced swallow *safety* (as identified in both FEES and CA assessments), the following characteristics were prevalent CA descriptors indicating reduced swallow *safety*: a change in swallow sound quality or length, and a change in respiratory quality and/or respiratory rate, findings which are congruent with previous research [24–29]. When FEES identified silent aspiration, CA raters noted increased upper respiratory tract noise (such as gurgling, wheeze and/or wet/moist respirations) throughout the swallow sequence, as well as a change in post swallow respiration, a characteristic also supported by other researchers [25, 27, 28]. This data is significant in terms of clinical application. Should clinicians identify the aforementioned respiratory features (even without the patient coughing/throat-clearing) in combination with other characteristics listed in Supplementary Material 9 (CA Assessment Protocol), then the risk for reduced swallow *safety* (penetration/aspiration) is a concern and gold standard instrumental swallow assessments (e.g. FEES or Videofluoroscopic Swallow Studies, VFSS) are strongly recommended.

For swallow *efficiency* (pharyngeal residue as per FEES), this aspect was also accurately identified by CA raters. The presence of multiple swallows observed on FEES correlated with CA descriptions of “clearing swallows” or “multiple swallows indicative of residue and poor bolus transit”. Additional to these descriptors related to residue, a change in respiration (quality and rate) was often simultaneously noted. This included descriptions of changes in respiration rate indicating swallow-breathing discoordination, possible residue, a long apnoeic period, and/or an inability to sustain the apnoeic period, with multiple swallow attempts, and/or attempts at breathing throughout the swallow sequence, depending on the dysphagic swallow that was presented (see Table 2). Many of these characteristics/features have previously been identified and discussed within dysphagia research [25–28, 30–35]. Again, in terms of clinical application, should these aforementioned characteristics of reduced *efficiency* and residue be prevalent during a CA assessment (together with other atypical/abnormal respiratory-swallow characteristics from Supplementary Material 9), then referral to instrumental assessment is warranted.

Finally, cervical auscultation is to be regarded as an assessment tool to complement the clinical swallow examination (CSE) [2, 18, 36]. CA does not fit the definition of a screening tool, since it is not a quick, simple-to-use screening test that requires minimal training and/or judgement for use [37]. Clinically, CA is used as an *adjunct* to the CSE in both paediatric and adult dysphagia management, with research demonstrating that CSE + CA results in a more accurate assessment than using CSE alone [18, 24, 25]. Although there is controversy regarding the place of the CSE in relation to instrumental assessments, such as Videofluoroscopic Swallow Studies (VFSS) or Flexible Endoscopic Evaluation of Swallowing (FEES), such controversy is unnecessary. These evaluation methods assess different aspects of the swallow / overall oral intake ability and contribute to different, yet complementary ICF and patient management perspectives [38, 39]. The clinical swallow examination (with or without CA) is not meant to replace instrumental assessments, yet it is essential for evidence-based and person-centered dysphagia management to, (i) holistically evaluate a range of dysphagia signs and symptoms throughout a meal and within the person’s natural environment, (ii) review and regularly follow-up a person’s swallow strategies and success with oral intake, and (iii) together with instrumental assessment, provide optimal dysphagia management as per the WHO, ICF [38–40].

## Limitations and future research

The CA assessment guide was developed based on the best available evidence, stemming from (i) an unpublished assessment protocol used in earlier research, with high validity, (ii) enhanced with new qualitative data and new literature, and (iii) reviewed by internationally recognized clinical experts / researchers in field of CA. However, further research (e.g. an international Delphi study), is recommended to provide opportunities to refine the current CA assessment guide and produce greater international consensus and guidance. Further qualitative analyses using a larger sample size and range of respiratory-swallow samples would provide a stronger qualitative analysis and is warranted for future research. To further strengthen the validity and generalisability, a larger sample size with varied consistencies across the different IDDSI levels is also recommended.

Finally, further international collaboration and guidance is recommended to facilitate future clinical research focusing on larger scale implementation of the protocol to assess effectiveness and reliability in diverse patient populations. Standard CA training and certification programs for SLPs would be recommended to ensure evidence-based and high-quality CA and dysphagia management application, where CA is used as an essential adjunct and tool to enhance the accuracy of the clinical swallow examination.

## Conclusion

This paper extends our previous research [2, 3, 18] by presenting previously unpublished data, new qualitative results, and by providing an evidence-based CA assessment guide, with CA audio files and reference samples. The CA protocol identifies five critical aspects to CA assessment: (1) pre-swallow respiratory sounds, (2) swallow sounds, (3) number of swallows, (4) exhalation post-swallow, and (5) post-swallow respiratory sounds, with specific dysphagic characteristics described. By sharing this assessment framework and learning kit, we aim to support the evidence-based application of CA in clinical settings however emphasise the importance of standard CA training to ensure high reliability and accuracy when using CA as part of the CSE. While the protocol represents a significant advancement, further research is recommended to continually refine the protocol and produce greater international consensus and guidance.

## Supplementary Information

Below is the link to the electronic supplementary material.

Supplementary Material 1

Supplementary Material 2

Supplementary Material 3

Supplementary Material 4

Supplementary Material 5

Supplementary Material 6

Supplementary Material 7

Supplementary Material 8

Supplementary Material 9

## Data Availability

The datasets used and/or analyzed during the current study are available from the corresponding author on reasonable request.
