# Supplementary material for: Respiratory-swallow assessment protocol for adult dysphagia management
Source: BMC Res Notes. 2025 Nov 27;19:60. doi: 10.1186/s13104-025-07509-4 (PMC12903235; doi:10.1186/s13104-025-07509-4)
Supplement: Supplementary file 9 — Supplementary Material 9 [file 13104_2025_7509_MOESM9_ESM.pdf]

APPENDIX A.
Cervical Auscultation Protocol for Respiratory-Swallow Assessment

Respiratory-swallow sound descriptors and evidence for distinguishing normal and dysphagic swallow sequences are detailed below.

Assessment is categorised into:
1. Pre swallow respiratory sounds (breathing sounds)
2. Swallow sounds (which occur during the apnoeic period)
3. Number of swallows
4. Exhalation post swallow (+/- glottal release sound, which may not always be present)
5. Post swallow respiration sounds

***NB.** The following respiratory-swallow sequence assessment is an evidence-based guide to inform clinical practice; however, it does not replace practical CA training. For valid and reliable CA assessment, training requires both a theoretical and practical, simulation training approach* [3].

NORMAL SWALLOW CHARACTERISTICS	ATYPICAL/ABNORMAL (DYSPHAGIC) SWALLOW CHARACTERISTICS
**1. Pre swallow respiratory sounds (breathing)**	
Regular / even (tidal) inhalation-exhalation breath sounds (Normal respiratory rate 12-20 breaths/minute [24, 25]).	Uneven breathing.<br>Fast/irregular breathing/respiratory rate [24, 25, §].<br>Increased work of breathing [§].
Clear breath sounds [24, 25].	Wet/gurgly sounding quality [24, 25, §].<br>Wheeze - airflow sounds turbulent [24].<br>Noisy/irregular breathing pre-swallow [§].
Swallow most commonly initiated during the exhalation phase (E-E)* or after inhalation preceding exhalation (I-E)* [17, 41].	Swallow initiated during inhalation [42, 43], combined with other abnormal features below [§].
**2. Swallow sounds + apnoeic period**	
Swallow sounds are quick (< 1 sec) [24, 25, 44-46].	Longer swallow sounds (e.g. >2 sec), drawn-out and/or uncoordinated [25, 26, §].
Thin fluids = loud and distinct [25, 45, 46].	Dull (softer) swallowing sounds [25, §].<br>Not distinct / lack of ‘crispness’ to swallow sound, may sound like a squelch/gurgly sound [§].<br>Absence of swallow sounds.

Thick fluid swallows = softer than thin fluids, yet still distinct (≤ 1 sec) [45-47, 55]. Longer swallow duration than for thin fluids [48, 49, 50, 55].<br><br>*Note! Swallow sounds become longer as we age [45, 46, 26, 51].*	Thick fluid swallows = Dull / long swallow sounds, > 2 sec, uncoordinated [29, §].<br>Absence of swallow sounds.<br><br>Extraneous abnormal sounds [25, 27, §].<br>High frequency squelching or gurgling, high pitched sounds, effortful/strained sounds [25, §].<br>Wet/gurgly respirations between swallow sequences for one bolus [27, §].<br>Voicing/throat-clearing between swallow sequences (for singular bolus) coupled with wet/gurgly respirations (features suggestive of residue) [§].<br>Apnoeic period = long breath-hold or unable to sustain breath-hold during swallow sequence (particularly if coupled with inhalation and sounds suggestive of residue post swallow, as described above) [27, 28, §].
**3. Number of swallows**	
Thin fluids, 10ml = usually 1 swallow [13].	> 1 swallow (in addition to other dysphagic features: example, abnormal swallow sound quality and/or change in respiratory rate or quality) [26, 27, 30, 31, §].
Thick fluids, 10 ml = 1-2 swallows / bolus [13].<br>Extra “clearing” swallows with thick fluids are normal and indicate an intact sensory system.	> 2 swallows (in addition to other dysphagic features).<br>Multiple swallows (>2) of a single bolus [27, §] coupled with wet/gurgly breath sounds between and after swallow attempts [§].
**4. Exhalation post swallow (+/- glottal release)**	
Exhalation post swallow.<br>Glottal release sound (vocal folds open with expiration / subglottic air pressure) may or may not occur directly after swallow sounds [25, 44, 53].	Inhalation after the swallow sounds [33, 34, §], in addition to other dysphagic features: for example, abnormal swallow sound quality and/or change in respiratory rate or quality, such as wet respirations, fast respiratory rate or increased work of breathing [§]. Especially notable if the pattern changes over subsequent bolus trials [28, 32].
Note: although the glottal release is not always heard [52, 53], exhalation post swallow is most common [17, 32, 53].	Long breath-hold / apnoeic period, coupled with additional dysphagic features [25, 35, §]
**5. Post swallow respiratory sounds**	
Exhalation after the swallow (+/-glottal release sound) [17, 25, 32, 52, 54], unless sequential swallows with cup/straw drinking [41].	Inhalation (instead of exhalation post swallow) [33, 34, 42, 43, §]**.
Breath sounds should return to **clear/regular** breathing - same as before the swallow [25, 35, 51].	Uneven breathing, different from pre-swallow breathing [25, §].<br>Change in respiratory rate: increased, irregular or shallow breathing [25, §].

	Breath sounds with additional features: wet, gurgly or wheeze [25, §].<br>Increased upper respiratory tract noise, sounding loud/ raspy [§].<br>Effortful/laboured breathing [§].<br>Throat-clearing/coughing, particularly when coupled with other dysphagic features [25, 35, §].

§ Data from current study, Table 2.
For an example of respiratory-swallow sequences, see Appendix B: Respiratory-swallow reference samples and Additional (audio) files 1-8.

* **E-E** = Exhalation – swallow – Exhalation; **I-E** = Inhalation – swallow – Exhalation.
****NB!** Respiratory-swallow analyses need to be interpreted within the context of the person's diagnosis and condition. For example, for some respiratory compromised populations (chronic obstructive pulmonary disease or respiratory failure), inhalation post swallow may be expected/usual for that person. The clinician should therefore be mindful of an increased risk for a dysphagic swallow and/or aspiration over multiple boluses, such as over the course of a meal. Remember to consider the entire respiratory-swallow sequences (as detailed in the above five areas) and both (a) the combination of, and (b) number of normal/abnormal (dysphagic) features at each bolus trial. Consider these characteristics across several swallow trials of (i) the same bolus, (ii) increased bolus size, (iii) increased drinking/feeding rate, and (iv) increased oral intake and/or over an entire meal.

Summary of the normal respiratory-swallow sequence

- Breathing pre swallow = regular/even breathing. Clear breath sounds.
- Normal respiratory-swallow pattern is either:
  - inhale, short exhalation → [apnoea during] swallow → continue exhalation; or
  - inhale → swallow → exhale
- Normal swallow sounds are quick, distinct, followed by exhalation (+/- glottal release sound).
  - Apnoea occurs during the swallow
- Breathing post swallow = same as pre swallow (regular/even breathing with clear breath sounds).

Importantly, consider similarity or variance of the respiratory-swallow pattern across several swallow trials to highlight normal and/or atypical/abnormal respiratory-swallow sequences.

APPENDIX B.
Respiratory-Swallow Sound Reference Samples (to accompany the additional audio files)

The tables below provide a description of respiratory-swallow characteristics for the normal (Table 1) and abnormal/dysphagic (Table 2) reference samples, included as additional audio files (see supplementary files). When listening to the normal and abnormal respiratory-swallow sounds, headphones or earpieces are recommended to reduce background and environmental noise.

Respiratory-swallow patterns are described as per recommended components:
1. Pre swallow respiratory sounds (breathing sounds)
2. (During) swallow sounds
3. Number of swallows
4. Exhalation post swallow (+/- glottal release sound, which may not always be present)
5. Post swallow respiration sounds

Additional sounds (such as the person's pulse) are written in *italics* with *.

Table 1. Normal swallow sound descriptors (to accompany the additional audio files 1-4).

NORMAL SWALLOWS	Description	PAS[a]
**Thin fluids (IDDSI [b]-0)**		
1. normal swallow_5ml_IDDSI-0_46 yrs.wav	1. **Even breathing** before the swallow. Clear breath sounds<br>**Additional sound: pulse.*<br>2. Swallow sound is quick (<1 sec)<br>3. One swallow (within normal limits)<br>4. Glottal release directly after (i.e. exhalation post swallow)<br>5. **Even** breathing (clear breath sounds) after the swallow.	1
2. normal swallow_10ml IDDSI-0_46 yrs_new.wav	1. Clear and even breath sounds before the swallow.<br>**Additional sound: at 4-5 seconds fluid movement in the oral phase.*<br>2. Swallow is quick (<1 sec)<br>3. One swallow / 10mls = normal.<br>4. Exhalation / glottal release directly after swallow<br>5. **Clear and even** breath sounds after the swallow. No change from pre-swallow breathing pattern.	1
**IDDSI-2 fluids**		
3. normal swallow_10ml_IDDSI-2_46 yrs.wav	1. Clear even breathing before the swallow<br>2. Swallow sound – quick (<1 sec)<br>3. One swallow	1

	4. Exhalation + glottal release immediately after swallow<br>5. Even, clear breathing after.<br>**Additional sound: rubbing of the stethoscope on skin.*	
**IDDSI-3 fluids**		
4. normal swallow_10ml_IDDSI-3_46 yrs_new.wav	1. Clear breathing pre-swallow<br>2 & 3. Two swallows for bolus (typical for IDDSI-3)<br>3. First swallow is quick, immediate glottal release following<br>Clear breath sounds between swallows.<br>Second swallow = quick<br>4. Glottal release/exhalation immediately after each swallow<br>5. Even, clear breathing post swallows.	1

[a] Penetration-Aspiration Scale, as per [19].
[b] IDDSI, as per [21].

Table 2. Dysphagic swallow sound descriptors (to accompany the additional audio files 5-8).

DYSPHAGIC SWALLOWS	Description	PAS[a]
**Thin fluids**		
5. abnormal_10ml_IDDSI-0.wav	**Additional sound: first 1-3 seconds of audio clip = stethoscope placement (rubbing on skin).*<br>1. Pre-swallow breathing = unclear/wheeze<br>2&3. (8-14 sec) Swallow sounds x4 = long, drawn out, not distinct, squelch/squeezed fluid sounds<br>● 15-17 sec = inhalation, short exhalation, inhalation (gurgly) then swallow<br>● 17-20 sec = swallow (number 4)<br>● 20 sec: exhalation.<br>4. Variable breathing during swallows, with inhalation occurring post swallow at times.<br>5. Post swallow breathing is non-clear sounds, wet/gurgly. Post swallow breathing is also laboured (effortful).	8
6. abnormal_10ml_IDDSI-0.wav	1. Pre-swallow breathing = clear (exhalation before swallow)<br>2. Swallow sounds: at 6-8sec) swallow 1 starts (soft, not distinct), long drawn out swallow sound.<br>Note: no breathing/apnoeic period (holding breath for a long time)<br>● 8-14sec: long breath hold (soft glottal release?) Note inhalation post swallow!<br>3. Three swallow sounds, at 15 sec = second swallow with extraneous squelch sound. At 16-17 sec, swallow 3 = not distinct, abnormal extra sound, post swallow exhalation, throat-clear + cough	7

	4. Post swallow *inhalation* after first swallow, exhalation after 2nd and 3rd swallow however breathing quality between swallows = wet/gurgly.<br>5. Post swallow breathing = unable to assess, stethoscope removed secondary to coughing.	
**IDDSI-2 fluids**		
7. abnormal_10ml_IDDSI-2.wav	1. Quiet breathing pre-swallow<br>**Additional sound: 6-7 seconds – patient voice*<br>•7-8 sec: exhalation, 9-13 sec: holding breath, slight inhalation before the swallow<br>2. Swallow sound (at 14-15 sec) = quick. Long breath-hold after.<br>3. One swallow only<br>4. Exhalation after swallow though abnormal, forced-sounding exhalation (squeezing sound). Coughing.<br>5. Post swallow breathing not recorded due to coughing.	2-7
**IDDSI-4 fluids**		
8. abnormal_10ml_IDDSI-4.wav	**Additional sound: first 1-5 seconds: stethoscope placement (talking/voices in the background)*<br>1. Pre-swallow breathing = unclear / wheezy<br>2&3. Four swallows. Swallow sounds:<br>● 5-6sec: swallow 1 = quiet, not distinct, slight post swallow exhalation<br>● Swallow 2 = quiet, indistinct swallows + breath hold and inhalation post swallow *(+ *Additional sound: stethoscope sounds, rubbing on skin)*<br>● 12 sec: swallow 3 = quiet, at 18 sec swallow 4<br>4. Variable breathing pattern post swallow with inhalation occurring after some swallows. Breathing = non-clear breath sounds, gurgly between and after swallows .<br>5. Post swallow breathing = non-even, non-clear breath sounds, wet/gurgly.	3

[a] Penetration-Aspiration Scale, as per [19].
[b] IDDSI, as per [21].
